# Evidence for niche adaptation in the genome of the bovine pathogen *Streptococcus uberis*

**DOI:** 10.1186/1471-2164-10-54

**Published:** 2009-01-28

**Authors:** Philip N Ward, Matthew TG Holden, James A Leigh, Nicola Lennard, Alexandra Bignell, Andy Barron, Louise Clark, Michael A Quail, John Woodward, Bart G Barrell, Sharon A Egan, Terence R Field, Duncan Maskell, Michael Kehoe, Christopher G Dowson, Neil Chanter, Adrian M Whatmore, Stephen D Bentley, Julian Parkhill

**Affiliations:** 1 Nuffield Department of Clinical Laboratory Sciences, Oxford University, John Radcliffe Hospital, Headington, Oxford, OX3 9DU, UK; 2 The Wellcome Trust Sanger Institute, Wellcome Trust Genome Campus, Hinxton, Cambridge, CB10 1SA, UK; 3 The School of Veterinary Medicine and Science, The University of Nottingham, Sutton Bonington Campus, Sutton Bonington, Leicestershire, LE12 5RD, UK; 4 Institute for Animal Health, Compton Laboratory, Compton, Newbury, Berks, RG20 7NN, UK; 5 Dept. of Veterinary Medicine, The University of Cambridge, Cambridge, CB3 0ES, UK; 6 Institute for Cell and Molecular Biosciences, The Medical School, University of Newcastle upon Tyne, Framlington Place, Newcastle upon Tyne, NE2 4HH, UK; 7 Department of Biological Sciences, University of Warwick, Coventry, CV4 7AL, UK; 8 Centre for Preventative Medicine, Animal Health Trust, Newmarket, Suffolk, CB8 7UU, UK; 9 Veterinary Laboratories Agency, Weybridge, UK

## Abstract

**Background:**

*Streptococcus uberis*, a Gram positive bacterial pathogen responsible for a significant proportion of bovine mastitis in commercial dairy herds, colonises multiple body sites of the cow including the gut, genital tract and mammary gland. Comparative analysis of the complete genome sequence of *S. uberis *strain 0140J was undertaken to help elucidate the biology of this effective bovine pathogen.

**Results:**

The genome revealed 1,825 predicted coding sequences (CDSs) of which 62 were identified as pseudogenes or gene fragments. Comparisons with related pyogenic streptococci identified a conserved core (40%) of orthologous CDSs. Intriguingly, *S. uberis *0140J displayed a lower number of mobile genetic elements when compared with other pyogenic streptococci, however bacteriophage-derived islands and a putative genomic island were identified. Comparative genomics analysis revealed most similarity to the genomes of *Streptococcus agalactiae *and *Streptococcus equi *subsp. *zooepidemicus*. In contrast, streptococcal orthologs were not identified for 11% of the CDSs, indicating either unique retention of ancestral sequence, or acquisition of sequence from alternative sources. Functions including transport, catabolism, regulation and CDSs encoding cell envelope proteins were over-represented in this unique gene set; a limited array of putative virulence CDSs were identified.

**Conclusion:**

*S. uberis *utilises nutritional flexibility derived from a diversity of metabolic options to successfully occupy a discrete ecological niche. The features observed in *S. uberis *are strongly suggestive of an opportunistic pathogen adapted to challenging and changing environmental parameters.

## Background

*Streptococcus uberis *is a gram positive bacterium belonging to family Streptococcaceae, a diverse family of bacteria that encompasses species capable of commensal and/or pathogenic traits. Pathogenic streptococci cause a variety of disease states across a range of animal hosts as well as man. The zoonotic potential of streptococci normally considered pathogenic for animal species has been recently documented for *Streptococcus suis *[1] and *Streptococcus agalactiae *[2].

Phylogenetic analysis [3] placed *S. uberis *within the pyogenic cluster, a large grouping containing the human pathogens *Streptococcus pyogenes *and *Streptococcus dysgalactiae *subsp. *equisimilis*, the zoonotic *S. agalactiae *and a number of animal pathogens occupying diverse ecological niches including *S. dysgalactiae, Streptococcus equi, Streptococcus canis *and *Streptococcus iniae*.

*S. uberis *is commensal at many body sites and has been isolated from the skin, gut, tonsils and genital tract of asymptomatic cattle. Furthermore it can infect the bovine mammary gland and act as a major pathogen of the mammary gland causing the inflammatory disease, mastitis. Infection with *S. uberis *is one of the major causes of bovine mastitis worldwide [4-6] and the most common cause in the UK [7]. Procedures to control bacterial infection of the mammary glands of dairy cattle are based on limiting duration of existing infection and restricting exposure of potentially infectious material from one gland to another. These procedures have resulted in decreased transmission of infections due to certain bacterial species (*Staphylococcus aureus*, *S. agalactiae*) but have had little impact on the incidence of infection due to *S. uberis*. The failure of these measures to control intramammary infection due to *S. uberis *implies transmission from additional/alternate sources [8]. Typing of isolates from cases of mastitis also implies that *S. uberis *is not transmitted from reservoirs containing single outbreak strains as multiple bacterial types are often detected within a single herd. *S. uberis *is often detected in faeces and can also be isolated from the environment (pasture, bedding materials) populated by these animals [9,10]. However, survival of *S. uberis *in the environment is limited. A recent report from New Zealand, which operates a pasture-based dairy system where cattle are housed rarely if at all, showed that the organism survived in the environment for less than 4 weeks [11]. This implies that persistence in pasture is dependent on constant reintroduction, probably via faecal contamination. It is, therefore reasonable to conclude that a successful clone of *S. uberis *isolated from a mastitic mammary gland is able to colonise and increase in number within the ruminant gut, survive in environmental niches such as pasture or bedding in sufficient numbers to gain access to the mammary gland where it must replicate and avoid a number of host defence mechanisms. In addition to infection of the lactating mammary gland, *S. uberis *is also able to infect the involuted or dry gland [12]. In this niche the secretion in which the organism replicates and the range of host defences encountered differ markedly from those present during lactation [13].

Epidemiologically, *S. uberis *strain 0140J, the strain chosen for sequence determination, was placed within a major UK lineage, the clonal complex based around sequence type 5, of an ongoing MLST scheme [14]. As such, strain 0140J represents a typical UK isolate in terms of its ancestry. It is also among the most thoroughly characterised strains [15] that is pathogenic for both the lactating and non-lactating bovine mammary gland. Therefore it was deemed ideally suited to be the first strain of this species to be sequenced. The complete *S. uberis *genome provides insights into host-cell interactions and pathogenesis.

Since the completion of the first streptococcal genome [16] many comparative projects have centred upon the main species pathogenic for humans, namely *S. agalactiae *[17], *Streptococcus pneumoniae *[18,19] and *S. pyogenes *[20,21]. Such studies have indicated the pairing of significant levels of conserved gene content with considerable gene sequence heterogeneity. Additionally, the proportion and content of such genomes that was attributable to a variety of mobile genetic elements appeared considerable. Comparative genomics has recently enabled the scale of both inter and intra-species horizontal gene transfer to be realised, for example within the oral streptococci [22]. Intriguingly, the gene content of some streptococci also appears to have been augmented from non-streptococcal species with which they co-exist in discrete ecological niches [23,24]. It is against such a backdrop that analysis of pathogenic streptococci of veterinary significance can derive added value. The genome sequences of several other related streptococcal species, with different host ranges and disease associations are available for comparison. We utilized the genomes of *S. equi *subsp. *zooepidemicus *(*S. zooepidemicus*) [25]; a veterinary pathogen causing lower airway disease, foal pneumonia, endometritis, and abortion in horses, and hemorrhagic streptococcal pneumonia in dogs; and *S. pyogenes *(alternatively referred to as group A Streptococcus, GAS) [26]; responsible for a diverse number of diseases in humans, including pharyngitis, toxic shock syndrome (TSS), impetigo and scarlet fever, and the post infection sequelae, acute rheumatic fever (ARF). Comparisons with these related pathogens and their virulence determinants highlighted the components of the genome that distinguish it from these species, and genes that are important for the niche-adaptation and virulence of *S. uberis*.

## Results and discussion

### Comparative genomics

**Figure 1:**
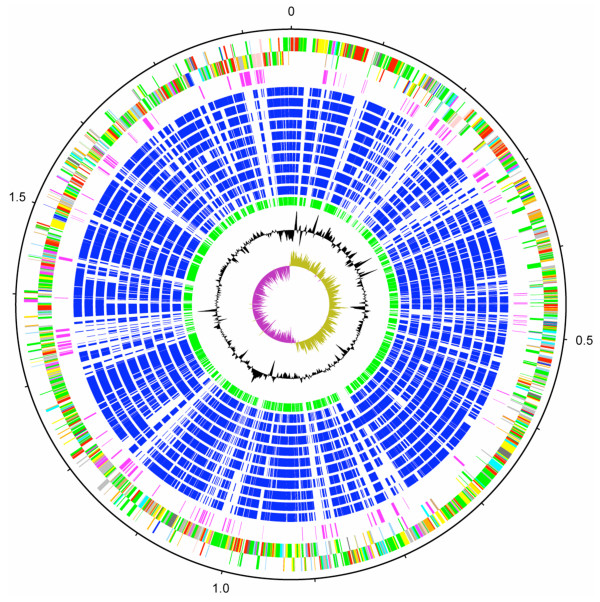
**Schematic circular diagram of the *S. uberis *0140J genome**. Key for the circular diagram: scale (in Mb); annotated CDSs coloured according to predicted function represented on a pair of concentric circles, representing both coding strands; *S. uberis *unique CDSs, magenta; CDSs with Streptococcal ortholog matches, blue; ortholog matches shared with the Streptococcal species, *S. pyogenes *Manfredo, *S. zooepidemicus *H70, *S. equi *4047, *S. mutans *UA159, *S. gordonii *Challis CH1, *S. sanguinis *SK36, *S. pneumoniae *TIGR4, *S. agalactiae *NEM316, *S. suis *P1/7, *S. thermophilus *CNRZ1066; *Lactococcus lactis *subsp. *lactis*, green; G + C% content plot; G + C deviation plot (>0% olive, <0% purple). Colour coding for CDS functions: dark blue; pathogenicity/adaptation, black; energy metabolism, red; information transfer, dark green; surface associated, cyan; degradation of large molecules, magenta; degradation of small molecules, yellow; central/intermediary metabolism, pale green; unknown, pale blue; regulators, orange; conserved hypothetical, brown; pseudogenes, pink; phage and IS elements, grey; miscellaneous.

The genome of *S. uberis *0140J consists of a single circular chromosome of 1,852,352 bp (Figure 1), which places it at the lower end of the 1.8 Mb–2.3 Mb size range of streptococcal genomes sequenced to date. The genome contains 1,825 predicted protein coding sequences (CDSs), 62 of which are pseudogenes or gene fragments (Additional file 1). Comparative genomic analysis with other streptococci by reciprocal FASTA revealed a conserved core of orthologous CDSs (Figure 1); comparisons using representatives of each of the sequenced *Streptococcus *species identified that ~40% of *S. uberis *CDSs had orthologous matches in all the streptococcal genomes compared. Supplementing this core were variably distributed orthologues (~48% of the CDSs) that were identified in one or more streptococci, and *S. uberis*-specific CDSs (~11% of the CDSs).

**Figure 2:**
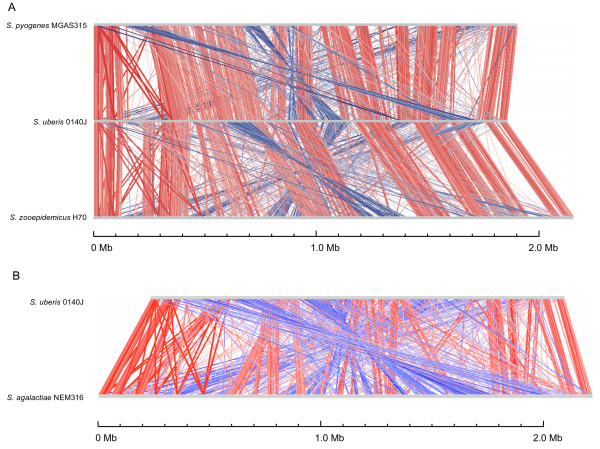
**Genome comparison of pyogenic streptococci**. Pairwise comparisons of the chromosomes of *S. pyogenes *MGAS315, *S. uberis *0140J and *S. zooepidemicus *H70 (A), and *S. uberis *0140J and *agalactiae *NEM316 (B) displayed using the Artemis Comparison Tool (ACT) [89]. The sequences have been aligned from the predicted replication origins (oriC; right). The coloured bars separating each genome (red and blue) represent similarity matches identified by reciprocal TBLASTX analysis [81], with a score cut off of 100. Red lines link matches in the same orientation; blue lines link matches in the reverse orientation.

For any one streptococcal species comparison, between 57% and 72% of the *S. uberis *CDSs had orthologue matches, which compared with 58% for a comparison with *Lactococcus lactis *subsp. *lactis*. The highest numbers of orthologue matches were identified in comparisons with pyogenic streptococci, while more taxonomically divergent species yielded lower numbers of orthologous matches. Comparison of the structure of the *S. uberis *0140J chromosome with other streptococci revealed the greatest overall conservation with *S. zooepidemicus *and *S. pyogenes *(Figure 2A).

Most of the conserved regions of these genomes appear to be co-linear, interspersed with regions that appear to be translocated and inverted. Of the currently sequenced streptococci, these two pyogenic species are also the most closely related to *S. uberis *as defined by 16S rDNA phylogeny [27]. A comparison with *S. agalactiae *revealed less conservation of genome structure (Figure 2B), suggesting a more distant genetic relationship.

In addition to the conserved regions identified in these comparisons, discrete regions of difference were identified throughout the genome of *S. uberis *0140J (Figure 1; Additional file 2), suggestive of diverse evolutionary origins for this component of the genome. Three discrete tracts of the sequence were identified as bacteriophage-derived islands, and a putative genomic island. When considered with additional remote CDS that are remnants of mobile genetic elements (MGEs), it was determined that MGEs constitute 1.7% of the genome. The low number of MGEs in the *S. uberis *genome is in marked contrast to other related streptococci [28,29]. Notably the genome does not contain any CDSs with similarity to insertion sequence (IS) elements.

**Figure 3:**
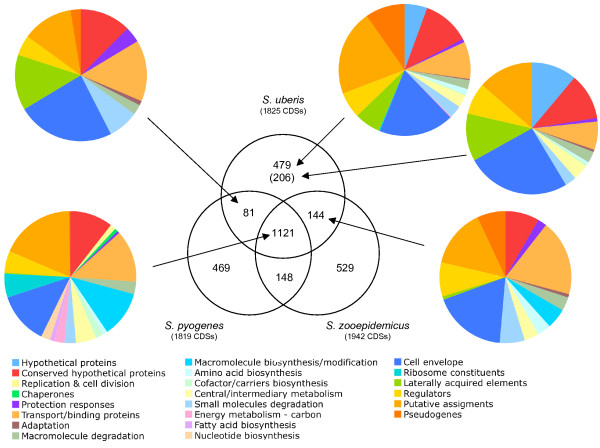
**Distribution of orthologous CDSs in *S. uberis *strain 0140J, *S. pyogenes *strain Manfredo and *S. zooepidemicus *strain H70**. The Venn diagram shows the number of predicted CDS unique or shared between one or more Streptococcal species. The figure in brackets indicates the number of unique CDSs that do not have ortholog matches in any of the other sequenced *Streptococcus *species. The relative distribution of functional groups of *S. uberis *0140J CDSs in the various sections is illustrated in the pie charts. Colour legend for the functional classification is below.

Whilst the genome comparison of *S. uberis *with other related pyogenic streptococci illustrates the common evolutionary origins of these species, it is apparent from the differences in host associations and pathogenicity that they have become specialized since they diverged from their common ancestor. Insight into the functional specialisations of the *S. uberis *genome can be gleaned from a tripartite comparison with *S. pyogenes *[26] and *S. zooepidemicus *[25] (Figure 3). The relative compositions of the differentially shared versus unique genome components exhibit differences that illustrate niche adaptation between the species. For example, the group of CDSs shared between *S. uberis *and *S. zooepidemicus *encodes functions associated with central metabolism, transport and gene regulation, which are absent in the group shared between *S. uberis *and *S. pyogenes*. In comparison to *S. uberis *and *S. zooepidemicus*, *S. pyogenes *is highly niche-restricted, therefore the spectrum of substrates and stimuli it experiences is narrower. This probably explains why *S. pyogenes *does not share the broader metabolic, transport and regulatory repertoire of the other two species. The *S. uberis*-specific group contains CDSs that differentiate this species from the other two pyogenic streptococci in this three-way comparison, but also distinguish *S. uberis *from other *Streptococcus *species (Figure 3).

Comparison of the relative compositions of the *S. uberis *vs. *S. zooepidemicus*-*S. pyogenes *unique CDSs (Figure 3) and the *S. uberis *vs. other streptoccoci unique CDSs (Figure 3) shows similar functional makeups to each other. The functions encoded in these groups encompass a diverse range including those associated with growth (central, catabolic and energy metabolism) and host- and environmental-interactions (transport, regulators, protective responses, and cell envelope), and reflect the potential for niche adaptation by *S. uberis*.

### Sugar utilization

In comparison to other streptococci, the *S. uberis *genome contains a distinct inventory of CDSs encoding carbohydrate degradation and utilization functions. The diversity of the sugar transport and utilization apparatus in the genome provides *S. uberis *with the capacity to survive in complex host and environmental niches. In particular, *S. uberis *is well equipped to utilize microbial metabolites and products arising from the digestion of plant material found in the rumen.

**Figure 4:**
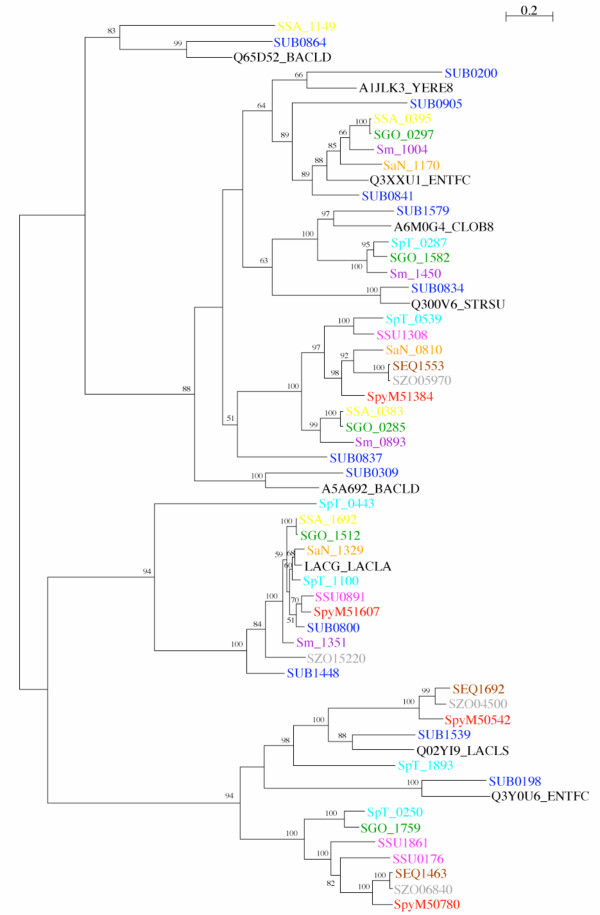
**Phylogenetic relationships of streptococcal glycoside hydrolase family 1 proteins**. Maximum-likelihood tree of streptococcal glycoside hydrolase family 1 proteins from: *S. uberis*, dark blue; S. *pyogenes *Manfredo, red; *S. equi *4047, brown; *S. zooepidemicus *H70 grey; *S. suis *P1/7, pink; *S. pneumoniae *TIGR4, *S. gordonii *Challis, green; light blue; *S. sanguinis *SK36, yellow; *S. mutans *UA159, purple; *S. algalactiae *NEM316, orange; black. Sequences of Fasta searches 'top match' hits in UniProt (Table 1), outside the streptococcal genomes used above have been included, black: Q3Y0U6_ENTFC, *Enterococcus faecium*; A1JLK3_YERE8, *Yersinia enterocolitica*; A5A692_BACLD, *Bacillus licheniformis*; Q300V6_STRSU, *Streptococcus suis*; Q65D52_BACLD, *Bacillus licheniformis*; Q3XXU1_ENTFC, *Enterococcus faecium*; LACG_LACLA, *Lactococcus lactis *subsp. *lactis*; Q02YI9_LACLS, *Lactococcus lactis *subsp. *cremoris*; A6M0G4_CLOB8, *Clostridium beijerinckii*. The tree was constructed with the WAG model of amino acid substitution, assuming a gamma distribution of among-site substitution rates. The numbers at the tree branches are percentage bootstrap values indicating the confidence levels, values below 50 are not shown.

*S. uberis *contains an expansion of glycoside hydrolase family 1 proteins [30] that catalyse the hydrolysis of glycosidic bonds between two or more carbohydrates, or between a carbohydrate and a non-carbohydrate moiety. The *S. uberis *0140J genome contains 12 members of a glycoside hydrolase family 1 (Pfam domain PF00232) in contrast to 4 in *S. pyogenes *Manfredo, 3 in *S. equi *4047, 4 in *S. zooepidemicus *H70, 4 in *S. suis *P1/7, 6 in *S. pneumoniae *TIGR4, 4 in *S. sanguinis *SK36, 4 in *S. mutans *UA159, 3 in *S. agalactiae *NEM316, 5 in *S. gordonii *CH1, and 6 in *Lactococcus lactis *subsp. *lactis *IL1403. The large number of glycoside hydrolase family 1 proteins suggests that *S. uberis *has the capacity to hydrolyse a wide range of sugars. Protein sequence similarity searches (Table 1) and phylogenetic analysis (Figure 4) demonstrates the diversity of the *S. uberis *proteins. The topology of the phylogenetic tree constructed with the glycoside hydrolase family 1 proteins suggests complex evolutionary origins of the proteins (Figure 4). For example, *S. uberis *SUB0800 is found in a clade containing a group of orthologous proteins from different streptococcal species (*S. pyogenes *SpyM51607, *S. zooepidemicus *SZO15220, *S. suis *SSU0891, *S. pneumoniae *SpT_1100, *S. sanguinis *SSA_1692, *S. mutans *Sm_1351, *S. agalactiae *SaN1329, *S. gordonii *SGO_1512) as well as SUB1448. The branch lengths of this clade suggest that SUB0800 is more closely related to most of the other streptococcal species proteins than to SUB1448, and therefore the likely ortholog. However, comparative genomic analysis examining synteny in the regions of the *S. uberis *loci with the other streptococcal members of this clade, identified SUB1448 as the orthologous protein rather than SUB0800. Analysis of the other glycoside hydrolase family 1 proteins shows that none of them can be identified as orthologues of glycoside hydrolase family 1 proteins in closely related streptococci on the basis of synteny. It is therefore not possible to resolve the complex evolutionary origins of the glycoside hydrolase family 1 proteins in *S. uberis *from the limited genomic datasets currently available. It is likely that expansion in the number of members of this family in *S. uberis *is the result of horizontal gene transfer, possibly from outside the genus. Database searches using the family members showed that for many of the proteins, the highest levels of amino acid identity are to proteins belonging to bacteria outside the genus. The most marked example is SUB0200, where the top Fasta matches are to proteins in Gram-negative enteric bacteria (Table 1).

**Table 1:** Homologues of the *S. uberis *glycoside hydrolase family 1 proteins.

***S. uberis *CDS**	**Protein**	**Organism**	**Identity**
SUB0198	Q3Y0U6_ENTFC	*Enterococcus faecium*	64.1%
	Q2BFH3_9BACI	*Bacillus *sp. NRRL B-14911	60.0%
	Q8ES64_OCEIH	*Oceanobacillus iheyensis*	60.3%

SUB0200	A1JLK3_YERE8	*Yersinia enterocolitica*	58.2%
	A4WDS3_9ENTR	*Enterobacter *sp. 638	56.8%
	Q6D574_ERWCT	*Erwinia carotovora *subsp. *atroseptica*	57.9%

SUB0309	A5A692_BACLD	*Bacillus licheniformis*	65.8%
	Q65MN0_BACLD	*Bacillus licheniformis*	65.8%
	Q3XWU5_ENTFC	*Enterococcus faecium*	64.2%

SUB0800	Q300R9_STRSU	*Streptococcus suis*	99.6%
	LACG2_STRPN	*Streptococcus pneumoniae*	92.9%
	A5M4I0_STRPN	*Streptococcus pneumoniae*	92.7%

SUB0834	Q300V6_STRSU	*Streptococcus suis*	80.9%
	O50658_9LACO	*Lactobacillus gasseri*	68.2%
	Q041B2_LACGA	*Lactobacillus gasseri*	68.0%

SUB0837	Q300W6_STRSU	*Streptococcus suis*	78.5%
	Q92F20_LISIN	*Listeria innocua*	61.6%
	Q8YA94_LISMO	*Listeria monocytogenes*	61.2%

SUB0841	Q839A6_ENTFA	*Enterococcus faecalis*	74.6%
	A3CKZ2_STRSV	*Streptococcus sanguinis*	73.6%
	Q8DU50_STRMU	*Streptococcus mutans*	72.2%

SUB0864	Q65D52_BACLD	*Bacillus licheniformis*	65.8%
	Q97TT6_CLOAB	*Clostridium acetobutylicum*	62.8%
	Q6D774_ERWCT	*Erwinia carotovora *subsp. *atroseptica*	62.1%

SUB0905	Q3XXU1_ENTFC	*Enterococcus faecium*	60.2%
	Q9CF10_LACLA	*Lactococcus lactis *subsp. *lactis*	59.6%
	Q3K0Z4_STRA1	*Streptococcus agalactiae*	58.6%

SUB1448	LACG_LACLA	*Lactococcus lactis *subsp. *lactis*	77.5%
	Q02V89_LACLS	*Lactococcus lactis *subsp. *cremoris*	77.3%
	LACG_STAHJ	*Staphylococcus haemolyticus*	76.8%

SUB1539	Q02YI9_LACLS	*Lactococcus lactis *subsp. *cremoris*	66.7%
	Q9CFI7_LACLA	*Lactococcus lactis *subsp. *lactis*	63.6%
	Q8XI15_CLOPE	*Clostridium perfringens*	59.0%

SUB1579	A6M0G4_CLOB8	*Clostridium beijerinckii*	68.1%
	A3UPR9_VIBSP	*Vibrio splendidus*	66.2%
	Q0KKN9_STALU	*Staphylococcus lugdunensis*	65.5%

Top 3 UniProt matches identified for each protein using FASTA.

Bacterial phosphoenolpyruvate-dependent sugar phosphotransferase systems (PTS) bind, phosphorylate, and transport sugar substrates across the bacterial cell membrane. In addition to performing sensing functions with respect to metabolite availability, components of PTS can also regulate metabolic and transcriptional processes [31]. Streptococci appear variously endowed with PTS, enabling uptake and growth on a range of carbohydrate energy sources, thereby equipping the respective species with the ability to survive and grow in a variety of ecological niches. *S. pneumoniae *(TIGR4) displays 21 PTS sugar-specific enzyme II complexes reflecting the utility of nutritional flexibility with respect to its host niche. Similarly, some fourteen PTS were identified in another oral streptococcus, *S. mutans *UA159 [32]. *S. uberis *can call upon an array of at least fifteen PTS to satisfy the requirements of fermentative or glycolytic energy production based upon the sugars available to itwithin the bovine gut and also in mammary gland secretions. Fifteen loci were identified in the 0140J genome that contained complete PTS, with an additional five loci containing components of PTS that may represent partial or divergent systems. In comparison, the non pathogenic dairy-industry bacterium, *S. thermophilus*, was reported to have seven PTS, of which four contain pseudogenes [23].

The *S. uberis *0140J genome contains a mannitol-specific PTS (SUB0288 and SUB0289) as part of an operon with a ribulose-phosphate 3-epimerase (SUB0285), 6-phospho-3-hexuloisomerase (SUB0286) and a mannitol-1-phosphate 5-dehydrogenase (SUB0287). These five CDSs do not have orthologous matches in the other streptococci. The metabolic genes in this cluster encode functions for conversion of D-ribulose 5-phosphate to D-xylulose 5-phosphate, isomerisation of hexulose-6-phosphate to fructose-6-phosphate and the production of D-fructose 6-phosphate from D-mannitol 1-phosphate.

Concomitant with its ability to colonise the bovine gut, the lumen of the mammary gland in lactating and non-lactating animals, and its ability to survive in pasture, *S. uberis *retains numerous regulatory CDSs. Many of the regulators in the accessory genome are associated with sugar detection and metabolism. These include 6 antiterminator type regulators associated with PTS (SUB0194, SUB0530, SUB0797, SUB0829 (a pseudogene), SUB1452, SUB1704), and 4 RpiR family regulators that contain SIS phospho-sugar binding domains (SUB0170, SUB0904, SUB1541, SUB1582)

### Energy metabolism

Within the CDSs unique to *S. uberis *when compared to *S. pyogenes *and *S. zooepidemicus *were two CDSs associated with energy metabolism (SUB0104 and SUB0105), that encode subunits of a cytochrome *d *ubiquinol oxidase. These CDSs are part of an operon (SUB0102 to SUB0107) similar to the respiratory chain operon of *S. agalactiae *(*menA*, *ndh*, *cydA, cydB, cydC*, and *cydD*) [33]. This respiratory chain is incomplete in *S. uberis*, as it is in *S. agalactiae*, as the genome does not encode the biosynthetic pathways for quinone, required for electron transfer, and haem, a cytochrome oxidase cofactor. However respiration in *S. agalactiae *can be stimulated under aerobic conditions if exogenous haem and quinone are supplied [33]. The presence of two distinct metabolic routes for energy production, fermentation and respiration, bestows *S. uberis *with a metabolic versatility that may promote survival in the diverse niches it occupies. *In vitro *experiments with *S. agalactiae *have shown a survival advantage for cells grown under respiratory conditions as opposed to under fermentation conditions [33]. Mutants of cytochrome *d *ubiquinol oxidase demonstrated lower levels of growth in blood under aerobic conditions, and also had reduced virulence in a neonatal rat sepsis model [33]. The ability to respire aerobically may be important for the spread and dissemination of *S. uberis*, although the requirement for exogenous haem and quinone suggest that this is strongly linked to environmental conditions dictated by the host or niche microbiota.

A recent study showed that quinones produced by *Lactococcus lactis *cross-feed *S. agalactiae *and activate respiration when the two organism were co-cultured [34]. Given the complexity of the microbial ecosystems in which *S. uberis *resides, it is not unreasonable to hypothesize that heme and quinone would be available permitting reconstitution of the respiratory chain. Cross-feeding of these key respiratory molecules by resident bacteria in the lower gastrointestinal tract may promote the fecal shedding of *S. uberis*. Whilst the anaerobic conditions of the gut may preclude respiration in this environment, once outside the gut it is possible that a shift in energy metabolism may promote growth and/or survival of *S. uberis *in fecal matter.

### Protective responses and environmental survival

The *S. uberis *genome encodes a polyphosphate kinase (SUB0262), a phosphate metabolism enzyme absent in other streptococci. This enzyme catalyzes the reversible transfer of the terminal phosphate of ATP to form a long-chain polyphosphate (polyP). The accumulation of polyP within *E. coli *cells has been shown to be a response to nutritional and osmotic stresses [35], and polyP has been demonstrated to be essential for long-term survival of *Shigella *and *Salmonella *spp. [36]. In *Vibrio cholerae*, polyphosphate stores enhance the ability of to overcome environmental stresses in a low-phosphate environment [37]. The presence of this enzyme in *S. uberis *suggests that this organism is equipped to tolerate comparatively low phosphate-availability environments such as those that might be encountered in faeces and pasture. Recent studies have also demonstrated a role for polyphosphate kinase in the pathogenicity of *Salmonella *and *Camylobacter *species [38,39].

Quaternary ammonium compound (QAC) disinfectants are used routinely as part of the everyday milking procedure in dairies as a hygiene measure. The *S. uberis *0140J genome contains a putative quaternary ammonium compound-resistance protein (SUB0162) that has 41% identity with QACH_STASA the quaternary ammonium compound-resistance protein QacH from *Staphylococcus saprophyticus *plasmid pST2H6 [40]. Downstream of this CDS there are two other CDSs that make a putative operon: SUB0163, binding-protein-dependent transport system membrane protein; and SUB0164, a fusion protein pseudogene. This latter CDS consists of an N-terminus similar to ABC transporter permease proteins and a C-terminus similar to fibronectin binding protein, containing an LPXTG motif. It is likely that this CDS arose due to a deletion event that resulted in the fusion of these functionally distinct domains. The three CDSs make up an operon, however it is not apparent what effect, if any, the mutation in SUB0164 will have upon resistance to QACs.

A distinct feature of the *S. uberis *genome that has emerged from the comparative genomic analysis with other streptococcal species is that there is an abundance of bacteriocins and associated processing, transport and immunity proteins. With the exception of the mammary gland, the niches that *S. uberis *colonize are highly competitive, and are populated by a diverse microbiota. Bacteriocins are proteinaceous antibiotics produced by bacteria that typically kill other bacteria of the same or closely related species. The production of bacteriocins by *S. uberis *is thought to provide a competitive edge and promote colonization.

Uberolysin is a novel cyclic bacteriocin produced by *S. uberis *(SUB0032 to SUB0036) [41]. It has been reported that some strains of *S. uberis *produce nisin U, a lantibiotic, which is similar to nisin A produced by *Lactococcus lactis *[42]. The 16 kb nisin U biosynthesis and resistance locus is absent in *S. uberis *0140J. The genome also contains a locus (SUB0502 to SUB0516) that encodes an array of bacteriocin structural proteins and immunity proteins. In total there are six CDSs encoding putative bacteriocin proteins (SUB0502, SUB0503, SUB0505, SUB0506, SUB0509 and SUB0512). The presence of multiple structural and immunity CDSs suggests a degree of redundancy. Several of the CDSs at this locus are pseudogenes (two of which are bacteriocin structural CDSs), suggesting that recent mutations have altered the bacteriocin expression repertoire of strain 0140J, and that there may be have been selection for the differential expression of CDSs at this locus. There is evidence of allelic variation at this locus in other strains of *S. uberis*; *S. uberis *strain E, contains a gene encoding (UbaA), a 5.3-kDa class IIa (pediocin-like) bacteriocin at this locus [43], whereas strain 0140J lacks *ubaA*, but contains divergent bacteriocin CDSs. Notably the ubericin A putative immunity protein UbaI is conserved in both strains (SUB0516; 98% identical at the amino acid level).

3.4% of the *S. uberis *0140J genome encodes pseudogenes (Additional file 1). Comparable levels are observed in the genomes of *S. suis *(strain P1/7) (unpublished data) and *S. pyogenes *(Manfredo) [26]; other Gram positive pathogens of similarly low GC content such as *Staphylococcus aureus *have a pseudogene content of between 0.8 and 2.5% [44,45]. These figures are significantly below the 10% figure reported for two *S. thermophilus *strains (CNRZ1066, LMG18311) for which disruption due to numerous mobile elements and an ongoing depletion of superfluous carbon utilisation CDS were deemed contributory factors [23]. Statistical analysis suggests that pseudogenes are over-represented in genomes of bacteria experiencing either new environmental niches or those becoming equipped to live in a diversity of habitats [45]. Although the relative level of pseudogenes is not high, it is a point of debate that the high representation of regulators and surface-associated proteins within this (Additional file 1), is suggestive of an organism engaged in the process of adapting to the challenge of successfully colonising the bovine gut and the mammary gland.

### Virulence

The majority of virulence factors in *S. uberis *genome are found in the variable component of the genome (Figure 3) although some are also found in the core gene set, for example fibronectin-binding protein (SUB1111) [46], hemolysin-like protein (SUB1273), and a C5A peptidase-like precursor (SUB1154). Overall there was a lack of what might be considered classical virulence determinants when compared with *S. pyogenes*. For example, genes associated with the production and surface anchoring of M protein in *S. pyogenes *are absent from *S. uberis *0140J. Orthologs of *emm *and *mga *(a transcriptional activator of *emm*) were not present and none of the CDSs identified previously as putative sortase substrates [47] suggested M-like properties. Other structural proteins that utilise sortase-mediated surface-anchoring such as pilin are also absent from the genome. As a pathogen, *S. zooepidemicus *has been less intensively studied, therefore the paucity of virulence determinants in this species, and consequently identified by comparison with *S. uberis*, reflects this. Detailed analysis of the *S. uberis *genome however identified several CDSs that have potential roles in virulence.

Pathogenic bacteria display an assortment of cell surface-associated proteins that can interact with host cells and tissues and contribute to the pathogenicity of the organism. Bacteria utilise a number of different mechanisms to attach proteins to the cell wall, one of which is through covalent linkage initiated by sortase enzymes. Up to 5 different classes of sortase enzymes have been identified in Gram positive bacteria [48,49] with one gene identified in the *S. uberis *genome, *srt*A (SUB0881). Anchoring of protein A of *Staphylococcus aureus *via SrtA has been well characterised [50]. Proteins that are destined for anchoring by sortase typically have N-terminal signal sequences for protein secretion and a cell wall sorting signal consisting of an LPXTG, or occasionally an LPX(A/S)G, anchoring motif followed by a C-terminal hydrophobic region with a positively charged tail of amino acids [51].

Seven genes were identified that contained LPXTG sorting motifs; SUB0145, SUB0164, SUB0348, SUB0888, SUB1095, SUB1730 and SUB1739 and a further two (SUB0207 and SUB0241) containing LPXAG motifs. However, two of these, SUB0164 and SUB1739, are highly likely to be pseudogenes and thus unlikely to be translated into fully functional, anchored proteins. A number of these genes have similarity with previously characterised streptococcal proteins; SUB0145, encodes a lactoferrin binding protein (Lbp) [52] which displays homology to fibrinogen-binding/M-like proteins, and SUB1095 shows similarity to an array of SclB collagen-like surface proteins of *S. pyogenes *[53]. Four additional CDSs, SUB0135, a fructan beta-fructosidase precursor and SUB0764, a surface-anchored 5'-nucleotidase (pseudogene), SUB0826, a subtilase family protein, and SUB1154, a C5A peptidase-like precursor, are also putative sortase-processed surface-anchored CDSs, containing the variant motifs LPXTS, LPXTS, LPXTR and LPXTV respectively.

A typical response to intra-mammary infection by the host is an increase in lactoferrin, an iron-binding glycoprotein that acts to restrict the availability of iron for bacterial growth [54,55]. Streptococci are considered to be resistant to the antibacterial effects of lactoferrin, most likely due to the low requirement for iron for growth [56]. However, the ability to sequester iron from host glycoproteins is considered to be essential for bacterial pathogenesis and bacterial iron-binding proteins have been investigated as potential virulence factors. Within the *S. uberis *genome both Lbp (SUB0145) and *S. uberis *adhesion molecule or SUAM proteins (SUB1635) have been investigated as potential iron-binding proteins [52,57,58]. The SUAM protein has an affinity for lactoferrin and is involved in the adherence of *S. uberis *to a bovine mammary epithelial cell line *in vitro *and therefore may be important during the initial stages of infection and colonisation [57,59]. Adherence experiments using *lbp *mutants however, suggest that Lbp is not required for attachment of *S. uberis *to host epithelial cells and it is also not essential for growth in iron-limiting conditions. The predicted protein sequence of Lbp is weakly similar (~28% identity) to the streptococcal M proteins, and the genome also contains a partial CDS (SUB0144) with homology to VirR12 and Mry which are positive regulators of M protein genes in group A streptococci [52]. The M protein and C5a peptidase are well characterised virulence determinants of *S. pyogenes *which act on different arms of the complement pathway, inhibiting bacterial opsonisation and inflammation and also phagocyte recruitment to the site of infection [60,61]. In *S. pyogenes*, these virulence determinants contain LPXTG motifs.

The hyaluronic acid capsule of *S. pyogenes *has been implicated in the pathogenesis of invasive GAS infections [62] and is essential for the maturation of GAS biofilms [63]. Strains of *S. uberis *isolated from cases of bovine mastitis display variable amounts of hyaluronic acid capsule. The complement and arrangement of hyaluronic acid biosynthetic genes in *S. uberis *differs from the *hasABC *arrangement common to GAS [64]. The *has *operon, consisting of *hasA *(SUB1697) encoding hyaluronan synthase and *hasB *(SUB1696) encoding UDP-glucose dehydrogenase, is similarly arranged to that in GAS. However a *hasC *homologue (SUB1691), encoding UDP-glucose pyrophosphorylase, was identified downstream of *hasB *in the reverse orientation and separated from it by some 3 kb containing CDSs apparently unrelated to capsule biosynthesis. The *hasA *gene product is essential for capsule production in both *S. pyogenes *and *S. uberis *[64,65]. However, capsule production in GAS is dependent only upon functional *hasA *and *hasB*, but not *hasC *[66]. The implication of functional redundancy for UDP-glucose pyrophosphorylase activity in GAS was further supported by the identification of additional homologous CDS in multiple GAS genomes. In contrast, lesions in isogenic acapsular *S. uberis *0140J mutants were confined to the *hasA *and *hasC *open reading frames suggesting no redundancy existed for these enzymes [64]. Correspondingly, a homologue (SUB1027) of the *S. uberis hasB *gene product (SUB1696) was identified that might account for the apparent inability to isolate a *hasB *mutant using phenotypic screening methods [64].

The lesions identified in non-mucoid mutants of *S. uberis *0140J are distributed between genetic loci likely to be involved in biosynthesis and also regulation. Three independent acapsular insertion mutants mapped within a putative GntR-like transcriptional regulator SUB0978 (unpublished data) suggestive of a role in upregulation of capsule precursors. The exploitation of an isogenic mutant lacking functional HasA demonstrated that the hyaluronic acid capsule of *S. uberis *plays little role in the early stages of infection of the lactating bovine mammary gland, and resistance to phagocytosis was ascribed to an undefined component unconnected with the capsular phenotype [67].

Activation of host plasminogen to plasmin, a potent serine protease that can bind to the surface of numerous bacterial pathogens, is a mechanism thought likely to augment a variety of streptococcal infections. Many of the streptococci within the pyogenic cluster express one of a variety of plasminogen activators that associate with the respective host's plasminogen to form an activation complex capable of recruiting and activating further substrate plasminogen to plasmin. The considerable sequence diversity, various substrate specificities and origins of the streptococcal plasminogen activators remain subjects of considerable research interest. The overwhelming majority of *S. uberis *isolates encode PauA (SUB1785) located between the *hexA *and *hexB *genes which display homology to the highly prevalent *mutS *and *mutL *DNA mismatch repair genes [68]. A single *S. uberis *isolate lacking *pauA *encoded an alternative novel plasminogen activator (PauB) [69] also at the *hexAB *locus.

An alternative locus in all human isolates of group A, C and G streptococci is populated by streptokinase (SK) [70]. The genes flanking SK did not appear to encode related functions, being an ATP-binding cassette protein and a leucine rich protein lying upstream of *ska*, while genes encoding a homologue of the *E. coli relA *and *spoT *genes which moderate levels of guanosine 5',3' polyphosphate during nutritional stress lie downstream [71]. The corresponding SK locus in *S. uberis *0140J is devoid of plasminogen activator CDS.

Some clues concerning the origins of the diversity of plasminogen activator sequences and the variety of genomic loci within which they appear in pyogenic streptococci are drawn from the observation that in at least three *S. pyogenes *serotypes (M1, M5 and M6) [26,72,73], prophage are located between the *hexA *and *hexB *orthologues with the phage attachment site for SF370.4 being mapped to overlie the start of the *mutL *coding sequence [72]. Further evidence of the role that phage play in generating variation at this locus is found in the CDS upstream of *pauA*, which encodes a putative exported protein (SUB1784), and is similar to proteins from *L. lactis *prophage.

Hyaluronidase, has long been considered to be a streptococcal virulence determinant due to its ability to break down the hyaluronic acid component of connective tissue and thereby facilitate spread of the pathogen, but there is little experimental evidence to support this assertion. Hyaluronic acid capsule produced by some streptococci, including *S. uberis*, is somewhat paradoxically also a substrate for hyaluronidase. Recently, it has been suggested that hyaluronidase facilitates the spread of high molecular weight compounds rather than bacterial cells *per se *[74] and also augments the effects of other streptococcal virulence factors such as pneumolysin [75]. In GAS, *hylA *encodes a large secreted hyaluronidase. In addition, numerous 'phage-associated' hyaluronidases (*hylP*) are evident. Neither GAS *hylA *or *hylP *homologues were present in the *S. uberis *0140J sequence. Interestingly the GBS2603 V/R locus encoding a chromosomal hyaluronidase (SAG1197) abuts *mutX *with the equivalent locus in GAS Manfredo being the prophage phiman.3, encoding one of a number of M5 phage hyaluronidase CDS. It appears highly likely that the hyaluronidase complement of streptococci has been and continues to be influenced by bacteriophage.

The CAMP factor [76], a further putative virulence factor homologous to the Fc-binding and haemolytic *cfb *gene product in *S. agalactiae *is absent from numerous strains of *S. uberis *[77]. A CDS encoding CAMP factor was not identified in *S. uberis *0140J or within 8 of a panel of 9 further pathogenic *S. uberis *strains tested (unpublished data) and can therefore be considered as non-essential to the infection of the bovine mammary gland.

## Conclusion

Analysis of the whole genome sequence of the bovine mastitis associated *S. uberis *strain 0140J has identified a relative paucity of classical streptococcal virulence determinants. In contrast, a plethora of metabolic options appear available to the bacterium. These endow it with the potential to flourish in a variety of nutritionally constrained habitats including the lumen of the mammary gland and the bovine gut. There are evidently survival advantages for an organism able to occupy multiple environmental niches. Critical to this aspect is nutritional flexibility, and the ability to derive carbon and energy by a variety of means. The genome provides compelling evidence supporting this proposal. Furthermore, *S. uberis *also appears equipped to cope with stresses likely to be encountered when shifting from one environmental niche to another for example through the use of polyphosphate kinase (SUB0262). We present here evidence that suggests *S. uberis *should no longer be regarded merely as a pathogen of the bovine mammary gland, but equally as a commensal microorganism of a wider bovine-based environment. Within this context, it will be of great interest to determine the current distribution of such features within the species and also whether *S. uberis *continues to refine or augment its genetic load along such lines in the future.

## Methods

### Strain

*Streptococcus uberis *0140J was isolated from milk obtained from a clinical case of bovine mastitis in 1972 within the UK. The strain *S. uberis *0140J has been deposited under ATCC^® ^Number BAA-854.

### Preparation of genomic DNA

Genomic DNA was prepared from *S. uberis *using a modification of the method of Hill and Leigh [78]. Todd Hewitt broth (400 ml) was inoculated with a single colony of *S. uberis *0140J picked from solid media and incubated for 18 h at 37°C. Bacterial cells were harvested, washed with 180 ml of Tris, 5 mM EDTA (pH 7.8) and the cell walls disrupted by incubation at 37°C for 30 mins in 50 ml of the same buffer, containing 30 u/ml mutanolysin and 10 mg/ml lysozyme. Cells were lysed by addition of 2.67 ml SDS (20% w/v in 50 mM Tris, 20 mM EDTA pH 7.8), 0.4 ml Proteinase K (20 mg/ml) and incubation at 37°C for 1 h. Saturated NaCl (26.7 ml) was added and after 5 min. cell wall material was removed by centrifugation for 20 mins at 16,000 × *g*. The supernatent was extracted twice with an equal volume of Tris-equilibrated (pH 8.0) phenol:chloroform:isoamyl alcohol (25:24:1). RNAase A was added to 20 μg/ml and the mixture incubated for 30 mins at 37°C. Genomic DNA was precipitated by addition of 2 volumes of ice-cold ethanol and incubation at 4°C for 2 h prior to centrifugation at 16,000 × *g *for 15 mins. The pelleted DNA was washed with cold 70% ethanol and air-dried prior to resuspension at 4°C in 2.5 ml of TE buffer.

### Whole genome sequencing

Sequence data were obtained from end sequences (giving approximately 8 × coverage) derived from M13 and pUC18 genomic shotgun libraries (with insert sizes of 1.4 to 2 kb and 2.8 to 3.3 kb respectively) using dye terminator chemistry on ABI3700 automated sequencers. End sequences from a large insert BAC library (pBACe3.6, 12 to 48 kb insert size) and Fosmid library (pEpiFos, 30–40 kb) were used as a scaffold. All identified repeats were bridged by read-pairs or end-sequenced polymerase chain reaction (PCR) products. The sequence was assembled and finished as described previously [79].

The sequence was annotated using Artemis software [79]. Initial coding sequence (CDS) predictions were performed using Orpheus [80], Glimmer2 [81], and EasyGene [82] software. These predictions were amalgamated, and codon usage, positional base preference methods and comparisons to the non-redundant protein databases using BLAST [83] and FASTA [84] software were used to refine the predictions. The entire DNA sequence was also compared in all six reading frames against UniProt, using BLASTX [83] to identify any possible coding sequences previously missed. Protein motifs were identified using Pfam [85] and Prosite [86], transmembrane domains were identified with TMHMM [87], and signal sequences were identified with SignalP version 2.0 [88]. rRNAs were identified using BLASTN [83] alignment to defined rRNAs from the EMBL nucleotide database; tRNAs were identified using tRNAscan-SE [89]; non-coding RNA (ncRNA) were identified using Rfam [90]. The sequence and annotation of the *Streptococcus uberis *0140J genome has been deposited in the EMBL database under accession number AM946015.

### Comparative genomics

Comparison of the genome sequences was facilitated by using the Artemis Comparison Tool (ACT) [91] which enabled the visualization of BLASTN and TBLASTX comparisons [83] between the genomes. Orthologous proteins were identified as reciprocal best matches using FASTA [84] with subsequent manual curation. Orthology inferred from positional information was investigated using ACT. Pseudogenes had one or more mutations that would prevent correct translation; each of the inactivating mutations was subsequently checked against the original sequencing data.

*Streptococcus *sequences used for comparative genomic analysis were: *S. pyogenes *Manfredo (accession number AM295007) [26], *S. pyogenes *MGAS315 (accession number AE014074) [92], *S. equi *4047 [25], *S. zooepidemicus *H70 [25], *S. thermophilus *CNRZ1066 (accession number CP000024) [23], *S. suis *P1/7 [93], *S. pneumoniae *TIGR4 (accession number AE005672) [94], *S. sanguinis *SK36 (accession number CP000387) [95], *S. mutans *UA159 (accession number AE014133) [32], *S. agalactiae *NEM316 (accession number AL732656) [96], and *S. gordonii *str. Challis substr. CH1 (accession number CP000725) [97], and *Lactococcus lactis *subsp. *lactis *IL1403 (accession number AE005176) [98].

### Phylogentic analysis

Unrooted maximum likelihood trees were built using PhyML [99,100] and drawn using NJplot [101]. Sequences were aligned using ClustalX (Version 1.82) [102]

## Abbreviations

ACT: Artemis Comparison Tool; ARF: acute rheumatic fever; CDS: coding sequences; GAS: group A Streptococcus; IS: insertion sequence; Lbp: Lactoferrin-binding protein; PolyP: polyphosphate; PTS: phosphotransferase system; QAC: quaternary ammonium compound; SK: streptokinase; TSS: toxic shock syndrome.

## Authors' contributions

PNW and SAE drafted the manuscript, MTGH annotated the genome and drafted the manuscript, JL conceived of the study, participated in the study design, and drafted the manuscript, MAQ and JW carried out the genomic library preparation, NL, ABi, ABa, LC, carried out the genome finishing and closure, DM and AW conceived of the study, participated in the study design and undertook critical review of the manuscript, TRF, MK, CD and NC conceived of the study, participated in the study design, BH, BGB, JP and SDB participated in the study design, managed the project and undertook critical review of the manuscript. All authors have read and approved the final manuscript.

## Supplementary Material

Additional File 1 **Table Pseudogenes S1: and partial genes in the *S. uberis *0140J genome.** Table S1: Table containing a list of the predicted products, systematic IDs, and putative mutations associated with pseudogenes and partial genes in the *S. uberis *0140J genome. Click here for file

Additional File 2 **Unique CDSs compared to other sequenced streptococci.** Table containing a list of the systematic IDs and predicted products for CDSs in the *S. uberis *0140J genome that do not have reciprocal Fasta matches in *S. pyogenes *Manfredo, *S. equi *4047, *S. zooepidemicus *H70, *S. thermophilus *CNRZ1066, *S. suis *P1/7, *S. pneumoniae *TIGR4, *S. sanguinis *SK36, *S. mutans *UA159, *S. agalactiae *NEM316, and *S. gordonii *str. Challis substr. CH1. Click here for file
