# Supplementary material for: Evidence for niche adaptation in the genome of the bovine pathogen Streptococcus uberis
Source: BMC Genomics. 2009 Jan 28;10:54. doi: 10.1186/1471-2164-10-54 (PMC2657157; doi:10.1186/1471-2164-10-54)
Supplement: Additional File 1 — Table Pseudogenes S1: and partial genes in the S. uberis 0140J genome. Table S1: Table containing a list of the predicted products, systematic IDs, and putative mutations associated with pseudogenes and partial genes in the S. uberis 0140J genome. [file 1471-2164-10-54-S1.pdf]

Additional file 1
Pseudogenes and partial genes in the *S. uberis* 0140J genome

Product	ID	Mutation type
response regulator protein	SUB0031	Nonsense mutation (ochre) after codon 152
bifunctional purine biosynthesis protein purH [includes: phosphoribosylaminoimidazolecarboxamide formyltransferase; IMP cyclohydrolase]	SUB0037A	Gene remnant
AraC family regulatory protein	SUB0096	Gene remnant
putative fusion protein	SUB0164	Fusion protein. No significant database matches to the full length CDS
UDP-glucose 4-epimerase	SUB0249	Frameshift after codon 17. Frameshift occurs at a direct repeat region (29mer)
putative membrane protein	SUB0272	Probable gene remnant. Missing C-terminal region contains transmembrane domain
type I restriction-modification system S protein	SUB0280	Frameshift after codon 389
putative dihydrolipoamide dehydrogenase	SUB0292A	Probable gene remnant
acetoin(diacetyl) reductase	SUB0293	Probable gene remnant
sensor histidine kinase	SUB0327	Probable gene remnant. a nonsense mutation (ochre) after codon 212
antiholin-like protein	SUB0327A	Probable gene remnant
putative surface-anchored protein	SUB0348	Probable gene remnant. CDS lacks an N-terminal signal sequence, therefore the protein is not likely to be processed
putative regulatory protein	SUB0361	Nonsense mutation (amber) after codon 32
putative membrane protein	SUB0461	Probable gene remnant
conserved hypothetical protein	SUB0476	Frameshifts after codons 43, 49 and 92
putative bacteriocin	SUB0502	Nonsense mutation (ochre) after codon 43
putative membrane protein	SUB0504	Frameshifts after codons 8, 62 and 103
conserved hypothetical protein	SUB0510A	Probable gene remnant
putative membrane protein	SUB0511	Frameshift after codon 2
ammonium transporter family protein	SUB0589	Nonsense mutation (ochre) after codon 35
putative membrane protein	SUB0611	Probable gene remnant resulting from a deletion. Frameshift after codon 11
putative membrane protein	SUB0612	Probable gene remnant resulting from a deletion. N-terminal region of the CDS is SUB0611
conserved hypothetical protein	SUB0653	Frameshift after codon 12. Frameshift occurs at a poly G pentamer
putative surface-anchored 5'-nucleotidase	SUB0764	Nonsense mutation (ochre) after codon 28
C-5 cytosine-specific DNA methylase	SUB0790	Frameshift after codon 72. Frameshift occurs at a poly G pentamer. CDS lacks an appropriate translational stop codon 72
conserved hypothetical protein	SUB0790A	Probable gene remnant
conserved hypothetical protein	SUB0804	Nonsense mutation (amber) after codon 88
putative integrase	SUB0805	Frameshift after codon 6. Frameshift occurs at a poly A nonamer
transcription antiterminator	SUB0828	Frameshift after codon 32. Frameshift occurs at a poly A hexamer
conserved hypothetical protein	SUB0887	Probable gene remnant
putative galacturonic acid acetylase	SUB1035	Probable gene remnant
CAAX amino terminal protease family protein	SUB1052	Nonsense mutation (opal) after codon 86
putative membrane protein	SUB1064	Nonsense mutation (opal) after codon 75
putative chromate transport protein	SUB1067	Nonsense mutation (ochre) after codon 183
conserved hypothetical protein	SUB1089	CDS lacks an appropriate inframe translational start site. Frameshift after codon 1
cation transporting ATPase	SUB1099	Probable gene remnant
conserved hypothetical protein	SUB1170A	Probable gene remnant
putative ABC transport/processing ATP-binding protein	SUB1171	Nonsense mutation (ochre) after codon 217
relaxase	SUB1178	Probable gene remnant

putative transposase	SUB1178A	Probable gene remnant
putative DNA recombinase	SUB1186	Possible gene remnant
putative sugar transporter	SUB1208	Nonsense mutation (amber) after codon 237
penicillin-binding protein 2b	SUB1259	Frameshift after codon 2. CDS lacks and appropriate in frame start codon
conserved hypothetical protein	SUB1340	Probable gene remnant
conserved hypothetical protein	SUB1346	Probable gene remnant. CDS lacks an appropriate stop codon
transposase	SUB1438A	Frameshift after codon 38 and a nonsense mutation (ochre) after codon 85. CDS lacks an appropriate stop codon
sugar phosphotransferase system (PTS), IIC component	SUB1440	Probable gene remnant. Nonsense mutations after codon 86 (ochre) and 247 (opal) and lacks appropriate start and stop codon
putative lactose-specific phosphotransferase system (PTS), IIBC component 1	SUB1450	Frameshift after codon 13 and a nonsense mutation (ochre) after codon 280. Frameshift occurs at a poly T heptamer
putative mannose-specific phosphotransferase system (PTS), IIC component	SUB1483	Frameshift after codon 61
3-hydroxybutyryl-CoA dehydrogenase	SUB1522	Probable gene remnant
putative citrate lyase beta subunit	SUB1587	Nonsense mutation (ochre) after codon 225
putative Mg2+/citrate transporter	SUB1594	Probable gene remnant
sensor histidine kinase	SUB1599	Probable gene remnant
putative transposase	SUB1600	Probable gene remnant
putative plasmid replication protein	SUB1608	Frameshift after codon 105. Frameshift occurs at a poly A pentamer
putative DNA-binding protein	SUB1609	Lacks an appropriate stop codon, possible frameshift in the C-terminal region could generate a CDS of the correct size
putative sensor kinase	SUB1672	Frameshift after codon 157
sensor histidine kinase	SUB1677	Nonsense mutation (opal) after codon 28, and a frameshift after codon 299. Frameshift possibly occurs at a poly T nonamer
putative surface-anchored protein	SUB1739	Nonsense mutations after codons 386 (ochre) and 436 (ochre)
putative DNA-binding membrane protein	SUB1798	Nonsense mutation (ochre) after codon 159
putative thioredoxin	SUB1807	Frameshift after codon 6
conserved hypothetical protein	SUB1817	Possible gene remnant
