# Supplementary material for: Evidence for niche adaptation in the genome of the bovine pathogen Streptococcus uberis
Source: BMC Genomics. 2009 Jan 28;10:54. doi: 10.1186/1471-2164-10-54 (PMC2657157; doi:10.1186/1471-2164-10-54)
Supplement: Additional File 2 — Unique CDSs compared to other sequenced streptococci. Table containing a list of the systematic IDs and predicted products for CDSs in the S. uberis 0140J genome that do not have reciprocal Fasta matches in S. pyogenes Manfredo, S. equi 4047, S. zooepidemicus H70, S. thermophilus CNRZ1066, S. suis P1/7, S. pneumoniae TIGR4, S. sanguinis SK36, S. mutans UA159, S. agalactiae NEM316, and S. gordonii str. Challis substr. CH1. [file 1471-2164-10-54-S2.pdf]

Additional file 2
Unique CDSs compared to other sequenced streptococci.

ID	Product
SUB0031	response regulator protein (pseudogene)
SUB0032	putative bacteriocin
SUB0033	putative membrane protein
SUB0035	ABC transporter ATP-binding protein
SUB0036	putative membrane protein
SUB0037A	bifunctional purine biosynthesis protein purH (fragment)
SUB0049	putative membrane protein
SUB0051	hypothetical protein
SUB0052	putative membrane protein
SUB0058	putative membrane protein
SUB0064	hypothetical protein
SUB0096	AraC family regulatory protein (fragment)
SUB0100	putative membrane protein
SUB0147	prophage maintenance system killer protein
SUB0149	hypothetical protein
SUB0153	conserved hypothetical protein
SUB0159	conserved hypothetical protein
SUB0162	putative quaternary ammonium compound-resistance protein
SUB0164	putative fusion protein (pseudogene)
SUB0167	conserved hypothetical protein
SUB0193	hypothetical protein
SUB0198	putative beta-glucosidase
SUB0199	6-phosphogluconate dehydrogenase family protein
SUB0200	6-phospho-beta-glucosidase 1
SUB0201	putative gluconokinase
SUB0208	putative membrane protein
SUB0209	hypothetical protein
SUB0211	putative membrane protein
SUB0212	putative exported protein
SUB0216	hypothetical protein
SUB0217	hypothetical phage protein
SUB0219	hypothetical phage protein
SUB0224	hypothetical phage protein
SUB0225	hypothetical phage protein
SUB0227	hypothetical phage protein
SUB0228	putative DNA-binding phage protein
SUB0229	putative DNA-binding phage protein
SUB0230	putative phage membrane protein
SUB0238	putative membrane protein
SUB0262	polyphosphate kinase
SUB0263	Ppx/GppA phosphatase family protein
SUB0272	putative membrane protein (fragment)
SUB0283	conserved hypothetical protein
SUB0285	putative ribulose-phosphate 3-epimerase
SUB0286	putative 6-phospho-3-hexuloisomerase
SUB0287	putative mannitol-1-phosphate 5-dehydrogenase
SUB0288	sugar phosphotransferase system (PTS), IIA component
SUB0289	putative mannitol-specific phosphotransferase system (PTS), IIBC component
SUB0292	hypothetical protein
SUB0292A	putative dihydrolipoamide dehydrogenase (fragment)
SUB0293	acetoin(diacetyl) reductase (pseudogene)

SUB0295	putative membrane protein
SUB0296	ABC transporter, ATP-binding protein
SUB0297	putative membrane protein
SUB0298	putative membrane protein
SUB0299	putative membrane protein
SUB0300	conserved hypothetical protein
SUB0309	6-phospho-beta-glucosidase 2
SUB0315	haloacid dehalogenase-like hydrolase
SUB0324	haloacid dehalogenase-like hydrolase
SUB0327	sensor histidine kinase (fragment)
SUB0327A	antiholin-like protein (fragment)
SUB0342	putative exported protein
SUB0348	putative surface-anchored protein (pseudogene)
SUB0378	hypothetical protein
SUB0460	putative membrane protein
SUB0461	putative membrane protein (fragment)
SUB0463	putative exported protein
SUB0476	conserved hypothetical protein (pseudogene)
SUB0478	putative membrane protein
SUB0498	response regulator protein
SUB0500	putative membrane protein
SUB0508	putative membrane protein
SUB0510A	conserved hypothetical protein (fragment)
SUB0514	hypothetical protein
SUB0519	putative membrane protein
SUB0528	putative exported protein
SUB0611	putative membrane protein (fragment)
SUB0612	putative membrane protein (fragment)
SUB0613	hypothetical protein
SUB0624	hypothetical protein
SUB0652	glyoxalase/bleomycin resistance protein/dioxygenase superfamily protein
SUB0705	N-acetylmuramoyl-L-alanine amidase
SUB0790A	conserved hypothetical protein (fragment)
SUB0807	putative transcriptional regulator
SUB0808	pyridine nucleotide-disulphide oxidoreductase family protein
SUB0826	putative surface-anchored subtilase family protein
SUB0834	6-phospho-beta-glucosidase 3
SUB0840	putative transporter protein
SUB0887	conserved hypothetical protein (fragment)
SUB0888	putative surface-anchored protein
SUB0892	putative exported protein
SUB0900	putative oxidoreductase
SUB0904	rpiR-family regulatory protein
SUB0905	6-phospho-beta-glucosidase 6
SUB0915	hypothetical protein
SUB0933	putative exported protein
SUB0934	hypothetical protein
SUB0987	hypothetical protein
SUB1013	putative membrane protein
SUB1022	conserved hypothetical protein
SUB1028	putative nucleotide sugar epimerase
SUB1030	putative acetyltransferase
SUB1034	putative glycosyl transferase
SUB1035	putative galacturonic acid acetylase (fragment)
SUB1052	CAAX amino terminal protease family protein (pseudogene)
SUB1057	conserved hypothetical protein

SUB1063	haloacid dehalogenase-like hydrolase
SUB1064	putative membrane protein (pseudogene)
SUB1067	putative chromate transport protein (pseudogene)
SUB1070	putative lipase
SUB1077	putative short chain dehydrogenase
SUB1083	conserved hypothetical protein
SUB1099	cation transporting ATPase (fragment)
SUB1137	putative membrane protein
SUB1160	CorA-like3 Mg2+ transporter protein
SUB1162	conserved hypothetical protein
SUB1170	conserved hypothetical protein
SUB1170A	conserved hypothetical protein (pseudogene)
SUB1171	putative ABC transport/processing ATP-binding protein (pseudogene)
SUB1177	putative membrane protein
SUB1178	relaxase (fragment)
SUB1180	putive copper chaperone CopZ
SUB1182	hypothetical protein
SUB1183	Crp family regulatory protein
SUB1184	hypothetical protein
SUB1185	puative DNA-binding protein
SUB1187	putative modification methylase
SUB1188	putative type III site-specific deoxyribonuclease
SUB1200	putative beta-glucuronidase
SUB1216	putative membrane protein
SUB1228	putative membrane protein
SUB1251	conserved hypothetical protein
SUB1252	hypothetical protein
SUB1336	Putative helicase
SUB1339	putative membrane protein
SUB1340	conserved hypothetical protein (fragment)
SUB1343	conserved hypothetical protein
SUB1345	putative acetyltransferase
SUB1349	MarR family regulatory protein
SUB1350	MATE family efflux protein
SUB1351	conserved hypothetical protein
SUB1353	putative amidase
SUB1360	NADH:flavin oxidoreductase / NADH oxidase family protein
SUB1361	3-dehydroquinate dehydratase
SUB1365	short chain dehydrogenase
SUB1366	conserved hypothetical protein
SUB1367	LysR family regulatory protein
SUB1400	hypothetical protein
SUB1401	hypothetical protein
SUB1448	6-phospho-beta-galactosidase 1
SUB1450	putative lactose-specific phosphotransferase system (PTS), IIBC component 1 (pseudogene)
SUB1451	putative lactose-specific phosphotransferase system (PTS), IIA component 1
SUB1510	CsbD-like protein
SUB1513	cold shock protein CspC
SUB1522	3-hydroxybutyryl-CoA dehydrogenase (fragment)
SUB1541	RpiR family regulatory protein
SUB1578	putative membrane protein
SUB1580	putative membrane protein
SUB1581	putative membrane protein
SUB1582	RpiR family regulatory protein
SUB1594	putative Mg2+/citrate transporter (fragment)
SUB1596	putative membrane protein

SUB1597	hypothetical protein
SUB1598	hypothetical protein
SUB1599	sensor histidine kinase (fragment)
SUB1602	putative sugar kinase
SUB1605	2-keto-3-deoxygluconate permease
SUB1606	4-deoxy-l-threo-5-hexosulose-uronate ketol-isomerase
SUB1607A	hypothetical protein
SUB1608	putative plasmid replication protein (pseudogene)
SUB1645	putative exported protein
SUB1646	putative membrane protein
SUB1649	hypothetical protein
SUB1674	sensor histidine kinase
SUB1675	putative sensor kinase
SUB1676	sensor histidine kinase
SUB1677	sensor histidine kinase (pseudogene)
SUB1687	putative exported protein
SUB1749	putative dioxygenase
SUB1750	conserved hypothetical protein
SUB1784	putative exported protein
SUB1785	PauA protein precursor (streptokinase precursor)
SUB1800	conserved hypothetical protein
SUB1801	putative coenzyme A transferase
SUB1803	putative fusion protein (3-hydroxyacyl-CoA dehydrogenase and enoyl-CoA hydratase/isomerase family protein)
SUB1804	acetyl-CoA acetyltransferase
SUB1805	putative acyl-CoA dehydrogenase
SUB1806	3-hydroxybutyryl-CoA dehydrogenase
SUB1807	putative thioredoxin
SUB1808	putative permease
SUB1809	ArsR family regulatory protein
SUB1817	conserved hypothetical protein (fragment)
SUB1819	hypothetical phage protein
SUB1820	hypothetical phage protein
SUB1821	hypothetical phage protein
SUB1822	hypothetical phage protein
SUB1823	hypothetical phage protein
SUB1824	phage membrane protein
SUB1825	hypothetical phage protein
SUB1827	hypothetical phage protein
SUB1829	hypothetical phage protein
SUB1830	hypothetical phage protein
SUB1834	hypothetical phage protein
SUB1835	hypothetical phage protein
SUB1839	putative DNA-binding phage protein
